# New Frontiers in Multiple Sclerosis Treatment: From Targeting Costimulatory Molecules to Bispecific Antibodies

**DOI:** 10.3390/ijms26083880

**Published:** 2025-04-19

**Authors:** Megan Reidy, Meerah Khan, Elizabeth A. Mills, Qi Wu, Josh Garton, Dean E. Draayer, Insha Zahoor, Shailendra Giri, Robert C. Axtell, Yang Mao-Draayer

**Affiliations:** 1 Autoimmunity Center of Excellence, Multiple Sclerosis Center of Excellence, Arthritis and Clinical Immunology Research Program, Oklahoma Medical Research Foundation, Oklahoma City, OK 73104, USA; megan-reidy@omrf.org (M.R.); meerah-khan@omrf.org (M.K.); joshua-garton@omrf.org (J.G.); draayded@gmail.com (D.E.D.); bob-axtell@omrf.org (R.C.A.); 2 Alzheimer’s Drug Discovery Foundation, 57 West 57th Street, Suite 904, New York, NY 10019, USA; elizabeth.ann.mills@gmail.com; 3 Department of Pharmaceutical Sciences, University of Michigan, Ann Arbor, MI 48109, USA; qiw@umich.edu; 4 Department of Neurology, Henry Ford Health, Detroit, MI 48202, USA; izahoor1@hfhs.org (I.Z.); sgiri1@hfhs.org (S.G.)

**Keywords:** multiple sclerosis, CD20, CD40L, CD19, CAR T cell, DMT, BTKi

## Abstract

Multiple sclerosis (MS) is an autoimmune demyelinating disease of the central nervous system. The therapeutic landscape for MS has evolved significantly since the 1990s, with the development of more than 20 different disease-modifying therapies (DMTs). These therapies effectively manage relapses and inflammation, but most have failed to meaningfully prevent disease progression. While classically understood as a T cell-mediated condition, the most effective DMTs in slowing progression also target B cells. Novel classes of MS therapies in development, including anti-CD40L monoclonal antibodies, CD19 chimeric antigen receptor (CAR) T cells, and Bruton’s tyrosine kinase (BTK) inhibitors show greater capacity to target and eliminate B cells in the brain/CNS, as well as impacting T-cell and innate immune compartments. These approaches may help tackle the disease at its immunopathological core, addressing both peripheral and central immune responses that drive MS progression. Another emerging therapeutic strategy is to use bispecific antibodies, which have the potential for dual-targeting various disease aspects such as immune activation and neurodegeneration. As such, the next generation of MS therapies may be the first to reduce both inflammatory demyelination and disease progression in a clinically meaningful way. Their ability to target specific immune cell populations while minimizing broad immune suppression could also lead to better safety profiles. Here, we explore the biological rationale, advantages, limitations, and clinical progress of these emerging immunotherapies for relapsing–remitting and progressive forms of MS.

## 1. Introduction

Autoimmune diseases occur when immune tolerance is lost. This is often a result of T cells and B cells becoming sensitized against antigens, resulting in autoreactive T cells and B cells that attack the host’s tissues [1]. The demyelinating autoimmune disease multiple sclerosis (MS) is the most common non-traumatic disabling disease among young adults. The typical pathology that characterizes MS is perivenular inflammatory lesions that result in demyelinating plaques. Historically, it has been classified as an organ-specific T-cell-mediated autoimmune disease. However, this view has been changed due to the success of B cell-targeted therapies [2].

The majority of MS patients experience the condition in two stages. The first stage of relapsing–remitting multiple sclerosis (RRMS), occurring in 85–90% of MS patients, is driven by focal inflammation, while the second stage of secondary progressive MS (SPMS) typically develops 10–20 years after disease onset and is driven by diffuse inflammation and neurodegeneration. About 10–15% of MS patients start with the progressive phase, referred to as primary progressive MS (PPMS) [2,3]. There are also patients that experience benign multiple sclerosis, in which the patients demonstrate little disease progression and minimal disability decades after disease onset. However, the number of cases show that benign multiple sclerosis is rare [4].

Inflammation results in oligodendrocyte damage and demyelination. Axons are preserved in the early stages of MS, but eventually, irreversible axonal damage occurs as the disease progresses. Focal inflammation during early MS (RRMS) is more pronounced and is associated with profound blood–brain barrier leakage, allowing inflammatory cells to infiltrate the brain from circulation. Thus, cortical and subcortical lesions formed in the early stages of MS have significant perivascular inflammation and diffusion of inflammatory cells into the parenchyma. Progressive MS has more diffuse innate microglia and astrocyte involvement with less lymphocytic infiltrates [5].

Acute lesions are composed of T cells, B cells, and macrophages. The immune profile of these lesions changes over the course of the disease, such that in progressive MS, the lesions are characterized by chronic active/smoldering lesions surrounded by a narrow rim of activated microglia and macrophages [2]. Microglia and macrophages release cytokines that can stimulate B cell activation and differentiation. B cells generate antibody and immune complexes which further activate microglia and macrophages. Patients with progressive MS also exhibit a relatively higher contribution of B cells and plasma cells [5]. This suggests that the contribution of different immune subsets may shift over the course of the disease, necessitating different therapeutic approaches.

Currently, there are successful treatment strategies for RRMS, such as interferons, sphingosine-1-phosphate receptor modulators, and monoclonal antibodies directed against either CD20, CD52, and a4-integrin, but these treatments have lacked efficacy in progressive MS, and can result in severe complications such as cytopenias, infection, or malignancy. Thus, there is an urgent need for identification of additional therapeutic targets [6].

B cells play a critical role in the pathology of MS, which was revealed in clinical trials using anti-CD20 monoclonal antibodies (mAbs), in which they were highly effective in preventing relapses. In MS patients, peripheral B cells display abnormal proinflammatory cytokine responses. Their depletion decreases the proinflammatory responses of CD4+ T cells, CD8+ T cells, and myeloid cells. B cells contribute to the pathogenesis of MS by presenting antigen to T cells, producing proinflammatory cytokines and chemokines, producing soluble toxic factors that injure neurons and oligodendrocytes, and memory B cells drive the auto-proliferation of brain-homing T cells. B cells can also help in the formation of ectopic lymphoid aggregates in the meninges, and may serve as a reservoir for Epstein–Barr virus [7].

The presence of oligoclonal bands (OCB), which are secreted, clonally restricted antibodies produced by B cells and plasma cells in the cerebrospinal fluid (CSF), are used as a diagnostic tool in MS patients. Their targets and role in MS pathology are still uncertain, and the search for autoantigens recognized by CNS antibodies continues. This is due to the fact that antibodies and complement are often deposited along myelin sheaths and on macrophage surfaces in active and newly forming MS lesions. Plasmablasts are an important source of these secreted antibodies, and they correlate with MS disease severity and gadolinium (Gd)-enhancing lesions [8]. Gd is a contrast agent that is a sensitive method for detecting active MS lesions, even though the correlation between enhanced lesions and clinical disease activity is only modest [9].

Anti-CD20 mAbs selectively interfere with the activation of B cells and their production of autoantibodies [10]. Pilot trials with rituximab demonstrated that even a single short course of an anti-CD20 mAb provides clinical benefits to patients with active rheumatoid arthritis [11]. However, rituximab did not show efficacy in trials for other autoimmune diseases in which B cells play a critical role, such as systemic lupus erythematosus (SLE) [12]. Rituximab was the first anti-CD20 monoclonal antibody treatment used in MS, followed by ocrelizumab, which was the first approved treatment used for RRMS and PPMS based on the outcomes in phase III clinical trials. Newer anti-CD20 antibodies are being improved through engineering techniques, such as humanization or fully humanizing the antibody to reduce immunogenicity and modifying the Fc region to enhance complement-dependent cytotoxicity and antibody-dependent cellular toxicity. More recently, ofatumumab and ublituximab were approved for RRMS [10,13,14,15,16].

An important limitation of these anti-CD20 mAbs is that they do not fully eliminate B cells. This is because effector cells that mediate antibody induced cellular toxicity, such as monocytes and natural killer cells, are not always residing in the tissues. Also, due to uptake through marginal zone macrophages, the anti-CD20 mAb concentrations in the tissues are lower, which decreases B cell clearance [1]. Studies have shown that there is more pronounced peripheral blood expansion of post-germinal center memory B cells than of marginal zone memory B cells in SLE patients that had shorter clinical responses post-treatment with rituximab, indicating incomplete tissue depletion of post-germinal center memory B cells. This incomplete depletion provides these post-germinal center memory cells the opportunity to selectively expand in their lymphopenic environment [17]. Also, stem cells (pro-B cells), many plasmablasts, and terminally differentiated antibody-producing plasma cells do not express CD20, and thus would not be eliminated with anti-CD20 mAbs. This incomplete depletion of B cells might be the underlying source of progressive disease activity [8].

**Figure 1:**
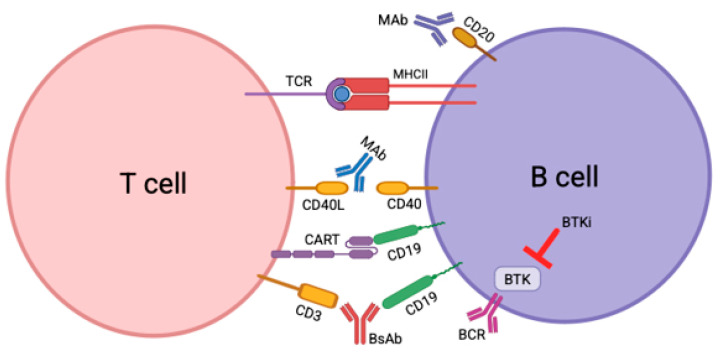
Upcoming therapeutics in multiple sclerosis. Anti-CD40L monoclonal antibodies, CAR T cells, and BTK inhibitors have been the most recently used in innovative research, showing promising outcomes for treating MS due to their abilities to target B cells and other innate cells in the CNS.

Due to these limitations, new methods of treatment have been developed with improved efficacy and safety for treatment of MS. This review will cover three new classes of potential therapies in treating MS, which are anti-CD40 ligand (CD40L) mAbs, chimeric antigen receptor (CAR) T cells, and Bruton’s tyrosine kinase inhibitors (BTKis) (Figure 1). Here, we explore the biological rationale, advantages, limitations, and clinical progress of these emerging immunotherapies for relapsing–remitting and progressive forms of MS. Another emerging therapeutic strategy is to use bispecific antibodies, which have the potential for dual targeting various disease aspects such as immune activation and neurodegeneration; however, they have not yet been used to treat MS.

## 2. CD40/CD40L

### 2.1. Preclinical Evidence of Target Validation

CD40 is a constitutively expressed membrane-bound costimulatory protein found on B cells and dendritic cells. Upon cell activation, the protein is also expressed by other cells such as T cells, monocytes, macrophages, endothelial cells, and CNS-resident cells [6]. CD40L is expressed on T cells and platelets, but it is also expressed on various other cells during inflammation, such as B cells, dendritic cells, macrophages, endothelial cells, and CNS resident cells. TNF receptor-associated factors (TRAFs) bind the cytoplasmic tail of CD40 to activate multiple signaling cascades. The CD40-CD40L dyad regulates the inflammatory phenotype of both immune and non-immune cells. Both CD4+ and CD8+ T cells can express CD40L during immune activation, but in patients with MS, CD40L expression is only detected on CD4+ T cells [6]. The cells expressing CD40 are in close proximity to cells expressing CD40L in the CNS of MS patients [18]. In healthy patients or in patients with other neurodegenerative disorders, CD40L has not been detected in the CNS, suggesting that CD40 and CD40L may be important drivers of inflammation in the CNS of MS patients [19,20]. In addition, CD40+ B cells have been identified inside inflammatory lesions of MS autopsy brain tissues, which suggests that T cell and B cell interactions mediated through CD40 pathway signaling could contribute to MS pathology [21]. Therapeutic antibodies have been developed targeting CD40L rather than CD40 due to its expression being relatively restricted to CD4+ T cells in MS [19]. Also, unlike anti-CD20 mAbs, anti-CD40L mAbs are able to penetrate the BBB, which shows promise in treating progressive disease activity [21].

Previous studies demonstrated evidence that B cells are not crucial to disease induction in experimental autoimmune encephalomyelitis (EAE) mice, which are the most common preclinical models of MS, and instead it was hypothesized that blocking the interaction between activated T cells with macrophages and microglial cells mediated the observed improvement in clinical symptoms in their EAE mice [19]. It was subsequently determined that the CD40-CD40L dyad is important in B cells in MS patients [18]. Stimulation of CD40 on B cells is important for their proliferation, differentiation, and survival, especially in the context of germinal center reactions, and it has been demonstrated that B cells from RRMS patients, but not healthy donors, are hyper-responsive to CD40 stimulation. Stimulation of CD40 activates canonical and non-canonical nuclear factor kappa B (NF***κ***B) signaling, MAP kinases, and phosphoinositide 3-kinase (P13K). CD40-induced canonical NF***κ***B pathway activation was found to be abnormal in B cells from RRMS and SPMS patients in comparison to healthy donors, while there was no significant difference in MAP kinase activation. Elevated levels of phosphorylated NF***κ***B in memory and naive B cells were also observed after CD40 stimulation in RRMS and SPMS donors were also observed in comparison to healthy donor controls. A significant positive correlation between EDSS and MFI values of phosphorylated NF***κ***B after CD40 stimulation has also been demonstrated in patients that received interferon beta-1a and combined mycophenolate mofetil combination therapy, indicating that reduced phosphorylation of NF***κ***B correlates with lower disease activity. Additionally, treatment with glatiramer acetate also significantly reduced NF***κ***B phosphorylation in RRMS patients. These data indicate that reducing CD40-mediated phosphorylation of NF***κ***B could improve clinical responses in MS patients [18].

### 2.2. Clinical Experience with Anti-CD40L

The safety and efficacy of multiple anti-CD40L drugs have been assessed in multiple autoimmune diseases. Ruplizumab (BG9588) was one of the first humanized anti-CD40L mAbs constructed to target the CD40 pathway [22]. CD40 expression is greatly upregulated in proliferative lupus nephritis, and several studies have demonstrated hyperexpression of CD40L by T cells and increased soluble CD40L (sCD40L) concentrations in lupus patients. In a phase II open-label study, the toxicity and efficacy of this drug was evaluated in patients with proliferative lupus glomerulonephritis. Serum complement concentrations and anti-dsDNA antibody titers were used as markers of biological activity, because CD40 and CD40L interactions are essential for the production of autoantibodies. A short course of treatment with BG9588 reduced anti-dsDNA antibodies and hematuria, increased C3 concentrations, and reduced spontaneously proliferating B cells. However, this study ended prematurely due to two cases of myocardial infarctions and due to serious thromboembolic events occurring in other BG9588 protocols [23].

Progressive MS is a debilitating demyelinating disease of the central nervous system (CNS) for which there are no effective treatments. Ending progressive MS is an urgent and unmet need. The inability to detect MS progression is also a major obstacle to prognostication, individualized therapies, and development of effective PMS treatments [24,25]. Using a novel approach to compare and contrast benign MS with progressive MS, our group discovered T cell costimulatory molecule CD40L as a predictive biomarker in MS disease progression [26]. This progressive MS biomarker discovery also links to potential new therapeutic discovery. Toralizumab (IDEC-131) was the first humanized anti-CD40L mAb assessed in MS [27]. The phase I clinical trial tested the safety and immune effects of blocking CD40L in MS patients. Toralizumab was administered to 12 patients with RRMS, who were monitored for up to 5 years. There were 15 mild-to-moderate adverse events, including gastric disturbances, fatigue, headaches, infections, and one case of non-disseminated herpes zoster (shingles), but no serious adverse events, such as thromboembolic events (Table 1). We were the first group to report that lymphocyte subsets were not depleted in response to anti-CD40L. In addition, we found an increase in CD25+/CD3+ and CD25+/CD4+ ratios as well as IL10/IL17 and IL10/MCP1 ratios, which indicates a shift towards an anti-inflammatory cytokine response; the increase in these ratios may be essential for the induction of tolerance [27]. These results led to further studies being conducted to assess the efficacy of blocking CD40L for treatment of MS.

### 2.3. Potential Safety Concerns

Although the several clinical studies with anti-CD40L mAbs had acceptable safety outcomes, the risk for thromboembolic complications is a potential concern. Thromboembolic complications were first observed in preclinical studies, and the occurrence of these adverse events in trials halted the clinical development of ruplizumab and toralizumab in lupus [22,23,28].

CD40 is constitutively expressed by the vascular endothelium of a variety of organs, while activated platelets express CD40L. Fresh thrombi have shown that CD40L was expressed on a significant number of platelets that were directly adhered to the endothelium and in areas where densely packed platelets had not yet formed a mass. It is possible that platelet activation and hypercoagulability were the result of the antibody forming immune complexes with the constant fragment (Fc) receptors on platelets and with sCD40L. However, whether these thromboembolic events were the result of blocking CD40L remains unclear. There have also been occurrences of thromboembolic events after treatment with a variety of antibodies besides anti-CD40L, but these events largely disappeared after refining the purification process of these antibodies [22,27].

There have been clinical trials discussed previously that reported no thromboembolic events after treatment with anti-CD40L. These differences in thromboembolic event outcomes could be due to how the antibodies were prepared, such as the potency of the antibodies and the manufacturing techniques. One study looked at the role of CD40 in CD40L and antibody-mediated platelet activation, and they proposed the mechanism that the Fc domains of monoclonal anti-CD40L antibodies form a complex with FcγRIIa on the surface of platelets that results in their clustering and stimulation, and possibly resulting in thrombosis. Thus, new drugs targeting CD40/CD40L need to address this concern [27,28,29].

### 2.4. Second Generation of CD40/CD40L Therapies in the Pipeline

The second-generation anti-CD40L mAb, frexalimab (SAR441344), was tested in a phase II double-blind randomized trial for relapsing MS (RMS) (NCT04879628). This antibody has been Fc-engineered to stop platelet activation as a result of FcγRIIa stimulation by immune complexes, which overcomes the risk of thromboembolic events. Frexalimab was well-tolerated and showed an 89% reduction in new gadolinium-enhancing (Gd+) T1 lesions in the high-dose arm vs. placebo at week 12. The number of Gd-enhancing T1-weighted lesions and new and enlarging T2-weighted lesions was consistently lower in patients that continued with frexalimab treatment in comparison to placebo controls at week 12, and the lesions decreased in a number in patients who switched from placebo to frexalimab treatment. The biomarkers neurofilament light (NfL), which indicates neuroaxonal damage, and CXCL13, which indicates inflammatory activity, decreased in patients who received frexalimab treatment. In the group of patients who received 1200 mg of frexalimab intravenously (i.v.), no relapses occurred during the 12-week double-blind period, and in the group of patients who received 300 mg of frexalimab subcutaneously (s.c.) and in the pooled placebo group, 4% had relapses during this period. These results showed an overall positive outcome for frexalimab treatment [30].

After week 12, 125/129 participants receiving placebo switched to respective frexalimab arms and entered the open-label extension (OLE). During the OLE, the s.c. dose was increased to 1800 mg q4w (which resulted in a similar exposure as with the 1200 mg q4w i.v. dose). Of these, 111 (89%) participants completed W96 with ongoing treatment. At week 96, 96% of participants in the high-dose arm were free of Gd+ T1 lesions, and this was 89% in those who switched from placebo to frexalimab at week 12. New/enlarging T2 lesion count and T2 lesion volume change remained low through week 96. No new safety signals were observed over 96 weeks of frexalimab treatment. The most common adverse events included COVID-19 and headache (in ≤10% of participants).

Larger and longer trials are needed to determine safety and efficacy for long-term treatment. Two currently ongoing phase III studies in both RRMS and non-relapsing secondary progressive (nrSPMS) are shown in Table 1; the latter is especially of unmet need. FREXALT (NCT06141473) includes FREXALT-1 and FREXALT-2 and is a double-dummy, active-controlled, event-driven 6-month composite-confirmed disability-worsening [cCDW] pooled across studies with variable treatment duration of approximately 20–40 months in RRMS participants. The planned enrollment is 1400 participants randomly assigned 1:1 to frexalimab or teriflunomide. Key eligibility criteria include RRMS diagnosis, Expanded Disability Status Scale (EDSS) ≤ 5.5, and ≥1 relapse within 1 year or ≥2 relapses within 2 years or ≥1 Gd+ lesion within 1 year. The primary endpoint is an adjudicated annualized relapse rate, and the key secondary endpoint is time-to-onset of 6-month cCDW (as assessed by the composite of EDSS, timed 25-foot walk and 9-hole peg tests).

**Table 1:** Recent and current interventional anti-CD40L clinical trials for MS.

Drug	Trial Phase	Trial Name or Number of Patients	Sponsor	MS Subtype	Recruitment Status	Primary Outcome	Outcome Met?	Secondary Outcome	Outcome Met?	NCT #
Toralizumab	I	12	Investigator-initiated/Biogen Idec Inc., Cambridge, MA, USA	RRMS	Completed	Adverse events up to 5 years after infusion Serum concentration of drug and anti-drug antibodies up to 18 weeks after infusion Immunologic analysis	Yes	Annualized relapse rate Total brain lesion volume Number of lesions at time of therapy induction and at 2 and 12 weeks post-therapy Total lesion load Maximum tolerated dose	Yes	N/A
Frexalimab	II	129	Sanofi, Paris, France	RRMS	Active, not recruiting	Number of new Gd-enhancing T1-hyperintense lesions at week 12	Ongoing	Number of new or enlarging T2 lesions at week 12 Total number of Gd-enhancing T1-hyperintense lesions at week 12 Adverse and serious adverse events until week 316 Anti-drug antibodies until week 316 Maximum concentration of drug until week 316 Time to maximum concentration of drug until week 316 Area under the curve over the dosing interval until week 316 Elimination half-life until week 316	Ongoing	NCT04879628
Frexalimab	III	FREVIVA:800	Sanofi, Paris, France	SPMS	Recruiting	Time-to-onset of composite confirmed disability progression up to 6 months after infusion	Ongoing	Time-to-onset of composite confirmed disability progression confirmed over 3 months up to week 204 after infusion Time-to-onset of individual components of the composite confirmed over 3 or 6 months up to week 204 after infusion Time-to-onset of confirmed disability improvement up to week 204 after infusion Number of new and/or enlarging T2 hyperintense lesions per MRI scan up to week 204 after infusion Percent change in brain volume loss as detected by MRI scans from week 24 in comparison to week 204 after infusion Change in cognitive function at week 204 in comparison to baseline as assessed by symbol digit modalities test Change from baseline in multiple sclerosis impact scale 29 version 2 questionnaire scores up to week 204 after infusion Change from baseline in patient reported outcome measurement information system fatigue multiple sclerosis-8a up to week 204 after infusion Annualized relapse rate Number of participants with adverse events up to week 204 after infusion Number of participants with potentially clinically significant abnormalities in lab tests, ECG, and vital signs up to week 204 after infusion Number of participants with antibody over time up to week 204 after infusion Change from baseline in serum immunoglobulin levels up to week 204 after infusion Change from baseline in plasma neurofilament light chain levels up to week 204 after infusion Drug plasma concentration up to week 204 after infusion	Ongoing	NCT06141486
Frexalimab	III	FREXALT: 700	Sanofi, Paris, France	RRMS	Recruiting	Annualized relapse rate	Ongoing	Time-to-onset of composite confirmed disability worsening over 3 and 6 months up to week 156 after infusion Time-to-onset of composite confirmed disability worsening over 3 or 6 months up to week 156 after infusion Time-to-onset of confirmed disability improvement up to week 156 after infusion Progression independent of relapse activity defined as the time-to-onset of 6 month composite confirmed disability worsening Total number of new and/or enlarging T2 hyperintense lesions as detected by MRI up to week 156 after infusion Total number of new Gd-enhancing T1 hyperintense lesions per scan as detected by MRI up to week 156 after infusion Percent change in brain volume loss as detected by brain MRI scans at the week 156 in comparison to week 24 Change in cognitive function at week 156 compared to baseline as assessed by the symbol digit modalities test Change from baseline in multiple sclerosis impact scale 29 version 2 questionnaire scores over time up to week 156 after infusion Change from baseline in patient-reported outcome measurement information system fatigue MS-8 up to week 156 after infusion Number of participants with adverse events leading to permanent study intervention discontinuation, AESIs, and safety scales up to week 168 after infusion Number of participants with potentially clinically significant abnormalities in lab tests, ECG, and vital signs up to week 168 after infusion Number of participants with anti-drug antibodies up to week 156 after infusion Change from baseline in plasma neurofilament light chain levels up to week 144 after infusion Drug plasma concentration up to week 144 after infusion	Ongoing	NCT06141473

FREVIVA (NCT06141486) is a placebo-controlled, event-driven cCDP trial with variable treatment duration of approximately 27–51 months in nrSPMS participants. Planned enrollment is 858 participants randomly assigned 2:1 to frexalimab or placebo. Key eligibility criteria include previous diagnosis of RRMS, current SPMS diagnosis, CDP within 1 year, EDSS 3–6.5, and no relapse within 2 years. The primary endpoint is time-to-onset of 6-month cCDP, and secondary endpoints include time-to-onset of 3-month cCDP and time-to-onset of 3-month/6-month CDP. Other secondary endpoints in both trials include clinical/MRI assessments, patient-reported outcomes, and safety evaluation. Inhibitors of the CD40 pathway could also down-regulate innate immunity in the CNS, which may help to treat progressive MS. The blockade of CD40L signaling on microglia at plaque edges in normal-appearing white matter, as well as on meningeal lymphoid follicles and macrophages, may combat compartmentalized inflammation [6]. MRI surrogates for microglial injury with slowly evolving and paramagnetic rim lesions (SEL, PRL), as well as possible PETs, are being evaluated as exploratory end points in ongoing phase III trials.

Multiple anti-CD40L or anti-CD40 drugs have been developed to reduce the risk of thromboembolic complications, such as dapirolizumab pegol (CDP7657) and VIB4920 in lupus, diabetes, Sjogren disease, and other autoimmune diseases.

## 3. CAR-T

### 3.1. Preclinical Evidence of Target Validation

CARs are engineered recombinant receptors for antigen, whose function is to redirect lymphocytes, most commonly T lymphocytes, to recognize and destroy cells expressing specific target antigens. When CAR-T cells bind to antigens on target cells, this results in vigorous T cell activation that is independent from MHC receptor binding (Figure 1). Other important features of CAR T cells are their ability to target cells that have down-regulated MHC expression or proteasomal antigen processing, and their ability to bind to carbohydrates and glycolipid structures (Figure 1). CD19 is the most investigated protein to target with CAR T cell therapy because it is present only in the B cell lineage and is found in most B cell lymphomas and leukemias. The first cancers to be eradicated by CAR T cell therapy were CD19+ malignancies, and, in 2017, the US Food and Drug administration (FDA) approved CD19 CAR T cell therapy for treatment of B cell malignancies [31,32]. Monoclonal antibody therapy has limitations in treating MS, which is why CD19-targeted CAR T cells are seen as a potential game changer. CD19 CAR T cells are seen as a superior option to monoclonal antibody therapy because the CAR T cells are able to penetrate the deep tissues, presumably including the CNS to kill the B cells in the brain, meninges, and spinal cord, whereas the monoclonal antibodies for CD19 and CD20 are too large to penetrate the BBB [33].

The anti-CD19 antibody MEDI551 depletes mature B cells, and it was shown to disrupt EAE via inhibition of multiple proinflammatory components without disrupting regulatory populations [34]. In a B cell-dependent EAE model that is responsive to anti-CD20 B cell depletion, CD19 CAR T cells extensively depleted B cells in the CNS and the surrounding periphery, and the depth of depletion was sustained beyond that observed with anti-CD20 monoclonal antibody treatment was also observed in this model. Clinical improvement was observed with CAR T cell treatment. However, there are various EAE models, and different models can show discrepancies in results when testing therapeutic drugs, which must be taken into account [35].

### 3.2. Clinical Experience of CAR T Cell Therapy in Cancer

Kymriah (tisagenlecleucel) was the first approved CD19 CAR-T therapy by the FDA for the treatment of relapsed/refractory pediatric and young adult B-cell acute lymphoblastic leukemia (ALL) in 2017 [36]. A phase I clinical trial in 2010 assessed the safety and feasibility of infusing the CD19 CAR T cells (CTL019) in patients with relapsed or refractory B-cell neoplasms. These CAR T cells addressed limitations in previous studies regarding tumor responses via incorporation of the CD137 (4-1BB) signaling domain, which promotes the persistence of these CAR T cells. In multiple-phase II studies, patients treated with tisagenlecleucel showed durable responses and acceptable safety profiles [37,38,39]. Since then, Yescarta, Tecartus, and Breyanzi have emerged as CD19 CAR T cell therapy treatments for various B cell malignancies [40,41,42].

### 3.3. Clinical Experience of CAR T Cell Therapy in Autoimmune Disease

More recently, a case series tested CD19 CAR T cell therapy in patients with severe, progressive autoimmune diseases, who were resistant to at least two different standard care immunomodulatory treatments. Fifteen patients received a single infusion of CD19 CAR T cells after preconditioning with lymphodepleting therapy drugs fludarabine and cyclophosphamide, and they were followed up to 2 years. Out of these 15 patients, 8 patients had SLE, 3 patients had idiopathic inflammatory myositis, and 4 patients had systemic sclerosis. After a mean of approximately 6 days post-treatment, CD19+ B cells were quickly eliminated from the peripheral blood of the patients. After approximately 112 days, B cells reappeared in 14 of the patients, and after 128 days, one of the patients still awaited B cell reconstitution. After 4 and 12 months post-treatment, the reconstituted B cells demonstrated a naive B cell phenotype, and CD19+CD27+ memory B cells were largely decreased [43].

The patients with SLE and idiopathic inflammatory myositis experienced complete resolution of disease symptoms, and patients with systemic sclerosis experienced decreased severity of lung and skin disease. These patients experienced full B cell reconstitution for at least 2 years without relapses, demonstrating that a single infusion of CD19 CAR T cells can result in long-lasting remission. The patients also experienced a sustained reduction in autoantibodies. While a role for the preconditioning chemotherapy treatment cannot be ruled out, it is highly unlikely that complete B cell depletion and sustained reduction in autoantibodies and drug-free remission were induced by the chemotherapy drugs based on results from previous studies. It is too soon to determine the durability of the treatment response, but this case series demonstrates that CD19 CAR T cells are capable of achieving sustained disease and drug-free remission for at least 2 years [43]. The success of CD19 CAR T cell therapy in various rheumatic and neuroimmunological diseases suggests possible future applications for MS.

### 3.4. Potential Safety Concerns

Although CAR T cells show great potential for treating progressive MS patients, currently only 20–50% of patients with lymphoid malignancies experience long-term remissions, which is why efforts must focus on designing new and improved CAR T cells for treating lymphoid malignancies [44]. There are still many limitations of CAR T cell therapy that must be addressed with every disease that the therapy is used to treat. Some of these limitations that are particularly important in regard to MS are antigen escape, on-target off-tumor effects, and CAR T cell-associated toxicities [32].

Antigen escape is the partial or complete loss of target antigen expression on the cells we seek to eliminate. A portion of the patients that respond well to CD19 CAR T cell treatment can later relapse with malignancies with no detectable CD19. The hypothesis for this mechanism is that there are CD19-spliced variants, and some of which lack the transmembrane domain of CD19, which results in loss of cell surface expression. During CD19 CAR T cell treatment, variants lacking cell surface expression can experience selective pressures that result in their proliferation [45]. In order to overcome this obstacle, CARs are now constructed with two distinct antigen recognition domains in order to target multiple antigens at once, which decreases the risk of selection of spliced variants that have no cell surface expression [32].

On-target off-tumor effects refer to the issue that the antigens we wish to target with CAR T cells are also expressed on normal tissues at various levels. To address this issue, CARs can be constructed to target antigens that have post-translational modifications that are specific to the targeted tumor. The effect of these types of modified CAR T cells are currently being investigated in colorectal cancer treatment [32].

CAR T cell-associated toxicities have been a significant barrier in making CAR T cell therapy a first-line treatment against cancers. The two most commonly observed toxicities are cytokine release syndrome (CRS) and immune effector cell-associated neurotoxicity syndrome (ICANS), which was previously known as CAR T cell-related encephalopathy syndrome (CRES). These toxicities can be fatal, which is why intensive monitoring and accurate assessment must be conducted with CAR T cell treatment. The toxicities that occur with CD19 CAR T cell therapy have been the most extensively characterized and studied to date [32,40].

CRS occurs in patients when T cells become extensively activated and release massive amounts of cytokines. Clinical symptoms present in patients as high fever, hypoxia, hypotension, and/or multi-organ toxicity [32,40]. In a study using CD19 CAR T cells to treat leukemia, all the patients experienced CRS, which was mild to moderate in 22 out of the 30 patients enrolled in the study. The patients experiencing mild to moderate CRS required hospitalization for febrile neutropenia. Severe CRS occurred in the other eight patients enrolled in the study, and the patients required varying degrees of respiratory support [46]. It is hypothesized that CRS is primarily mediated by IL-6, and so therapy options to mediate CRS toxicity involve blocking the IL-6 receptor, such as with tocilizumab, and involve the use of corticosteroids. However, death can still occur with treatment [32]. Rarely CRS can evolve into fulminant hemophagocytic lymphohistiocytosis (HLH), which is also known as macrophage activation syndrome (MAS). HLH/MAS is a severe hyperinflammatory syndrome that results in lymphohistiocytic tissue infiltration and immune mediated multi-organ failure [40].

ICANS occurs in patients when there are elevated CSF levels of cytokines that result in BBB disruption. Clinical symptoms present in patients as confusion, delirium, seizures, and cerebral edema [40]. In the study using CD19 CAR T cells to treat leukemia that was discussed previously, 13 out of 30 patients had neurologic toxic effects, and 6 patients had delayed encephalopathy that was independent of the severity of CRS and whether the patient was treated with tocilizumab [46]. The treatment for ICANS relies on corticosteroids [32].

Currently there are no approved therapies to prevent CRS, HLH/MAS, or ICANS. Thus, it is important to optimize CAR engineering and to use different strategies to decrease CAR-induced toxicities. Examples of improving upon CAR engineering include decreasing the affinity of the antigen-binding domain to decrease targeting of healthy tissues, using human-derived CARs instead of murine-derived CARs to decrease the likelihood of the host’s immune system reacting to the CAR, and adding an “off switch”, which essentially means constructing the CAR with mimotopes that can be bound with antibodies to inactivate the CAR. There have also been data that demonstrate that GM-CSF neutralization helps to reduce neurotoxicity, and there have been data that demonstrate that tyrosine hydroxylase inhibition results in decreased cytokine levels to help prevent CRS [32].

### 3.5. CAR T Cell Therapies in the Pipeline for MS

In 2024, a case report testing a fully human CD19 CAR T cell therapy (KYV-101) in two patients with progressive MS reported an acceptable safety profile. An enrichment of CD19 CAR T cells was observed in the CSF without resulting in neurotoxicity in the patients. However, one of the patients experienced CRS grade 1 a couple of hours after infusion. The drugs tocilizumab and dexamethasone were administered to prevent higher-grade CRS [3].

In progressive MS, meningeal B cell follicles continuously secrete cytokines and antibodies, and these follicles resemble tertiary lymphoid structures that are detected as isolated OCBs in the CSF. A reduction in OCBs was also observed, which was hypothesized to be due to a high expansion of CD19 CAR T cells in the CSF. This reduction in antibody production in the CSF was sustained through day 64 out of 100 days of follow up. However, the other patient had limited CAR T cell expansion. The investigators questioned whether ending peripheral B cell depletion before CAR T cell infusion requires an optimal effector-to-target ratio. This demonstrates the importance of the CAR T cells’ abilities to penetrate immune compartments that are not accessible to B cell-depleting monoclonal antibody therapy.

In addition to CD19, B-cell maturation antigen (BCMA) is another target that is currently being studied in phase I trials for MS and neuromyelitis optica (NMO) [47]. Progressive MS pathology could be due to long-lived plasma cells which do not express CD19 in high numbers, but they all express BCMA. These recent and current clinical trials are described in greater detail in Table 2. CAR T cell therapy is important for the future treatment of progressive MS because this therapy shows the potential to purge the CNS of the B cells that escape destruction by the monoclonal antibodies [3].

## 4. BTK Inhibitors

### 4.1. Preclinical Evidence for Target Evaluation

Bruton’s tyrosine kinase (BTK) is a protein that plays a crucial role in the signaling pathways of B cells and other cell surfaces (Figure 1). BTK is present in most hematopoietic cells, particularly in B cells, myeloid cells, and platelets, while T lymphocytes and plasma cells exhibit low or undetectable levels of BTK [48]. BTK plays similar roles in macrophages, microglia, and mast cells: it regulates their activation, promotes histamine release through degranulation in mast cells, and facilitates the secretion of proinflammatory cytokines and inflammasome activation [49]. BTK inhibitors (BTKi) are small molecules designed to treat B-cell malignancies by reducing B cell activity [50]. B cell activation causes chronic inflammation conditions, such as autoimmune disease symptoms [50]. The use of small molecule BTKi with good CNS penetrance could establish relevance in managing auto-inflammatory conditions, including MS.

A series of observations in human tissue has highlighted the significant role of BTK in MS, especially in the progressive forms of the disease. In brain autopsy samples from a patient with SPMS, the levels of BTK-expressing cells were elevated around the edges of the lesions [51]. This indicated the presence of BTK protein in microglia, with a significant rise in the concentration of BTK+CD68+ cells in the lesions compared to normal-appearing white matter [51]. Microglia play a crucial role in recruiting adaptive immune cells into the CNS [52]. This movement of lymphocytes into the CNS is a disease-related mechanism that could potentially be targeted using BTKi [52].

B cell activation is a consistent finding in MS, producing antibodies and stimulating other proteins that can cause damage, resulting in increased inflammation in MS patients [53]. In patients with MS, B cells may indirectly contribute to CNS damage by influencing the polarization of T helper cells both in the periphery and within the CNS. They also release various proinflammatory cytokines that activate macrophages and microglia, potentially facilitating the formation of follicle-like structures [54]. In MS brain tissue, the presence of these structures is linked to significant cortical demyelination and thinning, microglial activation, and the infiltration of T cells, macrophages, and some plasma cells into the CNS [54].

BTKi modify B cell functions but, unlike B cell depletion therapies, maintain B cell viability and survival [55]. Impairing the proper function of BTK decreases the activity of these immune cells, which have been associated with MS relapses [56]. Earlier studies have demonstrated that BTKi lead to a decrease in microglial expression, resulting in reduced levels of inflammatory surface markers [57]. This evidence postulates the effects of MS on microglia functions of demyelination and neurodegeneration [57]. This may be relevant for MS subtypes like PPMS and SPMS, where progression occurs independently of relapse activity and is partially driven by microglial activation, potentially serving as a significant factor.

### 4.2. Clinical Experience with BTKi

Most of the findings have arisen from studies on evobrutinib and tolebrutinib, both of which are covalent BTKi. Both evobrutinib and tolebrutinib reduce disease severity in a dose-dependent manner in both B cell-dependent and T cell-dependent EAE models [58].

Evobrutinib is an oral, CNS-penetrating inhibitor of BTK currently undergoing clinical development as a potential treatment for RRMS [48]. It is the first BTKi to show clinical efficacy in a large phase III study with follow-up extending beyond three years. Additionally, it has demonstrated an effect on early biomarkers of central inflammation linked to disease progression, including the volume of slowly expanding lesions (SEL) and levels of blood Nfl [59]. Subsequent studies in mice have shown that the efficacy of evobrutinib displayed a decrease in disease severity and significant improvement in meningeal inflammation.

Evobrutinib suppresses B cell activation and cytokine release, and modulates macrophage/microglia activation. In a randomized trial involving patients with RRMS, those treated with evobrutinib showed a significantly lower incidence of enhancing CNS lesions compared to those receiving a placebo [48,56]. Previous studies have demonstrated that evobrutinib treatment in mice with T cell-dependent EAE led to decreased leptomeningeal inflammation [59]. However, histological and immunohistochemical analysis revealed that evobrutinib treatment consistently reduced the proportion of B cells more than that of T cells, while the proportions of myeloid cells remained unchanged [59]. A recent study evaluating the safety and efficacy of evobrutinib versus teriflunomide found that evobrutinib’s efficacy was not superior to teriflunomide in terms of annualized relapse rate or any secondary endpoints, including disability and MRI measures of focal CNS inflammation [60]. It is not superior to teriflunomide regarding clinical, imaging, and biomarker endpoints, and does not have clinically significant effects on CNS-compartmentalized inflammation in individuals with RRMS [60]. Additionally, both study groups experienced a similar reduction in T1 gadolinium-enhancing lesions during the first 24 weeks. However, the total number of T1 gadolinium-enhancing lesions was numerically higher in the evobrutinib group compared to the teriflunomide group [60]. Overall, the efficacy and liver-related safety results do not support the use of evobrutinib in individuals with RRMS [60]. Results from ongoing phase III trials of other BTKi will provide insight into whether these findings can be applied to the entire class of BTKi.

Tolebrutinib is an oral, irreversible BTKi that penetrates the CNS [61]. This enzyme is expressed in B lymphocytes and myeloid cells, including microglia, which are key contributors to inflammation in MS [61]. Research has indicated that tolebrutinib performed well regarding safety, exposure, and pharmacodynamics in a phase I trial [62]. The drug was well tolerated by all participants, with no serious adverse effects reported, and the pharmacokinetic profile showed rapid absorption and favorable concentrations in the cerebrospinal fluid (CSF) [62].

Studies have shown tolebrutinib’s effects on tissue protection and repair. A mouse model study of cortical demyelination induced by recombinant antibodies from patients with MS and human complement components displayed that pretreatment with a tolebrutinib-like compound inhibited myelin degradation and the migration of microglia to demyelination sites [63]. It also showed the ability to prevent the loss of myelin and oligodendrocytes [63]. Subsequent studies in healthy volunteers have demonstrated that tolebrutinib remains at bioactive levels in the cerebrospinal fluid (CSF) four hours after administration of doses of 60 or 120 mg [64]. Other studies have indicated that tolebrutinib administration leads to changes in CSF protein levels in individuals with MS compared to untreated patients, further suggesting the presence of tolebrutinib in the CSF of those with MS [65]. The presence of BTK inhibitors in the CSF raises the possibility of relevant mechanisms within the CNS that warrant further investigation.

Unlike the recent evidence for evobrutinib, the latest findings for tolebrutinib have been more promising. In the HERCULES phase III study involving individuals with non-relapsing secondary progressive multiple sclerosis (nrSPMS), participants receiving tolebrutinib experienced a 31% delay in the time to the onset of 6-month confirmed disability progression (CDP) compared to those on placebo (HR 0.69; 95% CI 0.55–0.88; *p* = 0.0026). Further analysis of secondary endpoints revealed that the proportion of participants who achieved confirmed disability improvement nearly doubled, with 10% in the tolebrutinib group compared to 5% in the placebo group (HR 1.88; 95% CI 1.10 to 3.21; nominal *p* = 0.021) [66]. HERCULES is the first trial to demonstrate a notable reduction in disability progression among individuals with nrSPMS, a group with significant unmet needs.

GEMINI 1 and 2 phase III studies compared tolebrutinib with teriflunomide, a standard treatment, in participants with RRMS. Neither study achieved its primary endpoint of a statistically significant improvement in annualized relapse rates (ARR) when compared to teriflunomide. However, in a key secondary endpoint, a pooled analysis from GEMINI 1 and 2 indicated that participants receiving tolebrutinib experienced a 29% delay in the onset of 6-month confirmed disability worsening (CDW) compared to those on teriflunomide [66]. Tolebrutinib demonstrated a significant reduction in disability progression, despite no observed differences in relapse rates. These findings support the hypothesis that acute focal inflammation and chronic neuroinflammation are two separate biological processes. The combined data from HERCULES and GEMINI suggest that tolebrutinib consistently impacts disability progression, likely due to its effects on smoldering neuroinflammation. The long-term safety and efficacy of the studies mentioned are still being monitored. Additional evaluations will help confirm the continued efficacy of these treatments. Additionally, in the HERCULES study, liver enzyme elevations occurred in 4.1% of participants treated with tolebrutinib, compared to 1.6% in the placebo group. In the GEMINI study, liver enzyme elevations were noted in 5.6% of those receiving tolebrutinib, while 6.3% of participants on teriflunomide experienced similar elevations [66]. All cases were resolved without any lasting effects, and regular liver monitoring was implemented during the first 90 days.

Although showing promising results, tolebrutinib is still under investigation, and no definitive conclusions can be made about its safety and efficacy at this time without further FDA approval. 

### 4.3. Safety Concerns

Despite the promising results of BTKi use among study participants in MS trials, several adverse reactions have previously been linked to BTKi administration. Previous studies have shown that long-term use of BTKi have resulted in fungal infections, atrial fibrillation, ventricular arrhythmias, and sudden cardiac death, as well as BTKi resistance [67,68,69].

In a small trial for CNS lymphoma, 39% of patients treated with ibrutinib in combination with corticosteroids developed aspergillosis [70]. While antifungal prophylaxis is not warranted for the general population of patients being treated with ibrutinib, close follow-up and attention is recommended during the first initial months of treatment, especially for patients with high-risk features, such as diabetes, liver disease, and corticosteroid use [70].

Several randomized studies comparing BTKi to control therapies consistently showed a higher incidence of atrial fibrillation in patients treated with BTKi. Symptoms were shown to be manageable amongst patients, and do not need to be discontinued from BTKi treatments [68]. Study results suggest that patients being treated with BTKi should be closely monitored at each routine office visit during the first year of treatment initiation [68]. Sudden cardiac death has also been associated with BTKi treatment; however, this seems rare and associated with pre-existing comorbidities [68].

BTKi resistance has also been shown as a potential adverse effect in patients being treated for chronic lymphocytic leukemia (CLL) and non-Hodgkin lymphoma (NHL) [69]. Research has indicated that primary and acquired resistance to BTK inhibitors can occur through various mechanisms, including both intrinsic and extrinsic factors, such as gene mutations, activation of alternative signaling pathways, and the tumor microenvironment [69]. Further research is needed to evaluate the aforementioned adverse effects of BTKi treatment, as well as any other subsequent effects.

### 4.4. BTKi in the Pipeline

Tolebrutinib is undergoing continued clinical testing for its capacity to slow disease progression. The phase III, PERSEUS randomized, double-blind, placebo-controlled PERSEUS trial is currently assessing the safety and efficacy of tolebrutinib in participants with PPMS (NCT04458051). The primary outcome is the 6-month cCDP. These recent and current clinical trials are described in greater detail in Table 3.

Current ongoing investigations continue to test the efficacies of additional BTKi treatments, including orelabrutinib, fenebrutinib and remibrutinib. Orelabrutinib is currently being tested in a randomized, double-blind, placebo-controlled phase II clinical trial in patients with RRMS. Additionally, remibrutinib is also currently undergoing two phase III clinical trials, where the investigators are comparing the efficacy and safety of remibrutinib versus teriflunomide in patients with RRMS. An ongoing fenebrutinib phase III program includes two relapsing MS trials (including RRMS and active SPMS) as well as a PPMS study.

## 5. Bispecific Antibodies on the Horizon

Another avenue that could address the limitations of current DMTs is using bispecific antibodies (bsAbs), which are molecular biotherapies constructed to activate effector T cells and drive them to bind B cell antigens, which results in a similar cellular-dependent cytotoxicity as CD19 CAR T cells [71]. BsAbs recognize two different epitopes, and they vary in format, such as small proteins that consist of two linked antigen-binding fragments or large IgG-like molecules that have additional domains. The linkage of two binding specificities can result in a temporal or spatial dependency, in which the binding events occur either sequentially or simultaneously [72].

BsAbs can be constructed to be able to pass through the BBB, which is a huge limitation of using monoclonal antibodies as treatment for MS, such as anti-CD20. In order to engineer bsAbs to be able to pass through the BBB, one of the epitope-binding domains needs to bind to a BBB receptor, which would result in receptor-mediated transcytosis from the circulatory system into the brain parenchyma [73]. The other epitope-binding domain could be engineered to bind to MS targets of interest, like CD20, CD40L, or CD19.

In June 2024, the FDA approved blinatumomab (Blincyto) to treat patients with CD19-positive Philadelphia chromosome-negative B-cell precursor acute lymphoblastic leukemia [74]. The epitopes of blinatumomab are CD3 and CD19, and this drug demonstrated success as another T cell-activating strategy, similar in function as CD19 CAR T cells [72]. Unlike CD19 CAR T cells, bsAbs that are engineered to target CD19 have reduced severity of immune-related side effects, and they can be administered in a community setting without needing to make specific cellular products [71]. However, clinical trials will need to be conducted to determine safety and efficacy of this class of therapy, which as of now are on the horizon.

## 6. Conclusions

Anti-CD40L monoclonal antibodies, CAR T cells, and BTK inhibitors have been the most recent in innovative research, showing promising outcomes for treating MS due to their abilities to target B cells in the CNS. Fc engineering has made anti-CD40L mAbs a promising therapy without thromboembolic complications; phase III studies that are currently recruiting will determine its efficacy and safety for both RR and SPMS. CD19 CAR T cells are favored due to their ability to penetrate deep tissues and ability to target the CNS, but the side effects are still difficult to manage, and the requirement for certified tertiary care centers to be concentrated in urban centers, the production times, and the high per-patient costs could make the treatments unobtainable for many people [71]. The most recent findings of tolebrutinib treatments seem to be the most promising in treating progressive MS patients in phase III trials, however detailed analysis and results are awaiting to be reviewed for FDA approval. Future studies also need to test BTKis with less exposure to reduce side effects and/or drug resistance.

Many challenging factors need to be considered when deciding on treatment options for progressive MS, such as timing, safety, efficacy, availability, and cost. Each of these treatments has pros and cons, and hopefully future clinical trials will shed light on which treatment is best suited for patients, and/or if each of these treatments will work for progressive MS.

## Figures and Tables

**Table 2:** Recent and current interventional CAR T cell therapy clinical trials for MS.

Drug	Trial Phase	Sponsor	MS Subtype	Recruitment Status	Primary Outcome	Outcome Met?	Secondary Outcome	Outcome Met?	NCT #
KYV-101 (CD19 CAR T)	I	Kyverna Therapeutics, Emeryville, CA, USA	Non-relapsing, progressive	Recruiting	Frequency of dose-limiting toxicities for each dose level up to 12 months after infusion	Ongoing	Adverse events up to 12 months after infusion Drug concentration in the blood and CSF up to 12 months after infusion Level of B cells in blood up to 12 months after infusion Time from infusion to a change in disability and walking score as measured by EDSS Levels of unique unmatched intrathecal oligoclonal bands up to 6 months after infusion Whole brain and gray matter volume up to 12 months after infusion	Ongoing	NCT06138132
KYV-101 (CD19 CAR T)	I	Kyverna Therapeutics, Emeryville, CA, USA	Progressive	Recruiting	Drug concentration following peak expansion in peripheral blood up to week 48 after infusion Adverse events and dose-limiting toxicities up to week 48 after infusion	Ongoing	Proportion of patients with reduction in CSF OCB and/or normalization of CSF IgG index	Ongoing	NCT06451159
KYV-101 (CD19 CAR T)	II	Kyverna Therapeutics, Emeryville, CA, USA	Progressive	Recruiting	Confirmed disability progression as measured by EDSS	Ongoing	Adverse events up to 2 years after infusion Composite confirmed disability progression up to 12 weeks after infusion Drug levels up to 2 years after infusion Level of B cells in blood up to 2 years after infusion Level of serum cytokines up to 2 years after infusion Percentage of patients that developed anti-KYV-101 antibodies	Ongoing	NCT06384976
Universal BCMA CAR T, universal CD19 CAR T	I	Xuanwu Hospital, Beijing, China	Not listed	Not yet recruiting	Dose-limiting toxicities up to 28 days after infusion Adverse events up to 12 months after infusion	Ongoing	Concentration of drug in peripheral blood up to 3 months after infusion Level of B cells in peripheral blood up to 3 months after infusion Changes in pathogenic antibody titers in peripheral blood or CSF at 1, 3, 6, and 12 months after infusion Annualized relapse rate Changes in Myasthenia Gravis Activities of Daily Living (MG-ADL) score at 1, 3, 6, and 12 months after infusion Changes in Inflammatory Neuropathy Cause and Treatment (INCAT) score at 1, 3, 6, and 12 months after infusion Change in number of Gd-enhancing T1 lesions at 6 and 12 months after infusion Change in number of new or enlarging T2 lesions at 6 and 12 months after infusion	Ongoing	NCT06485232
C-CAR168 (CD20/BCMA-directed CAR T)	I	RenJi Hospital, Shanghai, China	RRMS	Recruiting	Adverse events and dose-limiting toxicities up to 3 years after infusion	Ongoing	Proportion of patients who achieved remission at 6 months after infusion Proportion of patients who achieved remission up to 3 years after infusion Proportion of patients who relapsed up to 3 years after infusion Time to response of first remission Progression-free survival up to 3 years after infusion Proportion of patients who achieved either low or no dose of glucocorticoids and/or immunosuppressants up to 3 years after infusion Maximum plasma concentration of drug up to 3 years after infusion Time to achieve maximum plasma concentration of drug up to 3 years after infusion Duration of drug in peripheral blood up to 3 years after infusion Area under the curve of drug in peripheral blood up to 3 years after infusion Clearance of peripheral blood B cells up to 3 years after infusion Decline of serum immunoglobulin up to 3 years after infusion Elevation of peripheral blood complement up to 3 years after infusion Decline of autoantibodies or other disease specific biomarkers up to 3 years after infusion	Ongoing	NCT06249438
CT103A (BCMA CAR T)	I	Tongji Hospital, Wuhan, China	RRMS	Recruiting	Dose-limiting toxicities up to 28 days after infusion Adverse events up to 2 years after infusion	Ongoing	Changes in concentration of soluble BCMA in peripheral blood after infusion up to 2 years after infusion Changes in pathogenic antibody titers in peripheral blood or CSF up to 2 years after infusion Changes in peripheral blood neurofilament light chain concentrations up to 2 years after infusion Number of BCMA CAR gene copies in peripheral blood and CSF up to 15 years after infusion Concentration of BCMA CAR T cells in peripheral blood up to 28 days after infusion	Ongoing	NCT04561557
YTB323 (CD19 CAR T)	I/II	Novartis Pharmaceuticals, Basel, Switzerland	RRMS	Not yet recruiting	Adverse events up to 2 years after infusion	Ongoing	EDSS score up to 2 years after infusion Short-form health survey to assess quality of life up to 2 years after infusion Timed 25-foot walk up to 2 years after infusion 9-Hole Peg Test up to 2 years after infusion Symbol Digit Modalities Test up to 2 years after infusion Fatigue symptoms and impacts questionnaire up to 2 years after infusion Number of new and enlarging T2 lesions and Gd-enhancing T1 lesions up to 2 years after infusion Maximum plasma concentration of drug up to 2 years after infusion Area under the curve of drug up to 2 years after infusion Time to reach maximum concentration of drug up to 2 years after infusion The last quantifiable plasma concentration of drug up to 2 years after infusion The time of the last quantifiable concentration of drug up to 2 years after infusion Humoral immunogenicity of drug up to 2 years after infusion Cellular immunogenicity of drug up to 2 years after infusion Drug meeting release specifications at or above target dose between day 9 and day 2	Ongoing	NCT06617793

**Table 3:** Current clinical development and characteristics of BTK inhibitors for MS.

Drug	Trial Phase	Sponsor	Trial Status	Study name and Total Number of Participants	MS Subtype	Primary Outcome	Outcome Met?	Secondary Outcome	Outcome Met?	NCT #
Tolebrutinib	III	Sanofi, Paris, France	Completed	GEMINI 1: 824 GEMINI 2: 762	RRMS compared to teriflunomide	Annualized relapse rate (ARR) (number of adjudicated MS relapses in a year)	No—Annualized relapse rate was low in the teriflunomide arm in both GEMINI 1 and 2 and no difference was observed between tolebrutinib and teriflunomide.	Time-to-onset of 6-month confirmed disability worsening (CDW)	Yes—tolebrutinib demonstrated clear separation from teriflunomide (29% relative risk reduction) in a population with very low relapse activity.	NCT04410978 (GEMINI 1) NCT04410991 (GEMINI 2)
								Time-to-onset of 3-month CDW	Yes—tolebrutinib demonstrated similar effects on time to 3-month CDW as 6-month CDW.	
								Time-to-onset of 6-month confirmed disability improvement	Yes—there was a numerically higher rate of 6-month confirmed disability improvement in the tolebrutinib arm compared to teriflunomide.	
								Total number of new Gd-enhancing T1 brain lesions	The number of Gd-enhancing T1 lesions was higher in the tolebrutinib arm.	
								Total number of new/enlarging T2 brain lesions	The number of new/enlarging T2 lesions was similar between both treatment arms	
								% change in brain volume	Brain volume loss (BVL) from month 6 to EOS was low for both tolebrutinib and placebo groups	
Tolebrutinib	III	Sanofi, Paris, France	Completed	HERCULES: 869	nrSPMS	6-month confirmed disability progression (CDP) [time frame: up to approximately 48 months]	Yes-Tolebrutinib showed a 31% risk reduction in time to 6-month CDP vs. placebo	Time-to-onset of 3-month CDP	Tolebrutinib demonstrated a significant effect on time to 3-month CDP	NCT04411641 (HERCULES)
								Time-to-onset of 6-month CDI	Proportionally more participants experienced CDI on tolebrutinib vs. placebo	
								Total number of new or enlarging T2 lesions	Tolebrutinib significantly lowered the annualized rate of new/enlarging T2 lesions vs. placebo	
								% change in brain volume	Brain volume loss (BVL) from month 6 to EOS was low for both tolebrutinib and placebo groups	
Evobrutnib	III	Merck KGaA, Darmstadt, Germany	Completed	EVOLUTION RMS1: 807 EVOLUTIONRMS2: 847	RMS	Annualized relapse rate	No—Evobrutinib did not show superior efficacy to that of teriflunomide in either study	Number of new T1 gadolinium-enhancing lesions	The total number of T1 gadolinium-enhancing lesions and adjusted mean number per scan were numerically higher for evobrutinib than for teriflunomide in both studies	NCT043380 (EVOLUTION RMS1) NCT04338061
								New or enlarging T2 lesions	The total number of new or enlarging T2 lesions was similar between groups in both studies and did not show an increase from week 24 in the evobrutinib group	
								Serum NfL concentration at week 12, ng/L	Adjusted geometric mean serum NfL concentrations at week 12 showed no difference between evobrutinib and teriflunomide in evolutionRMS1 and a nominal difference in evolutionRMS2. Prespecified exploratory analysis showed a similar reduction in serum NfL by week 96 from baseline between evobrutinib and teriflunomide in both studies, with no increase in serum NfL after week 24	
Evobrutinib	II	Merck KGaA, Darmstadt, Germany	Completed		RMS, RRMS	Total number of gadolinium-enhancing T1 lesions	Yes—patients with RMS who received 75 mg of evobrutinib once daily had significantly fewer enhancing lesions during weeks 12 through 24 than those who received placebo	Annualized relapse rate (ARR)	No—no significant difference with placebo for either the 25 mg once-daily or 75 mg twice-daily dose of evobrutinib, nor in the annualized relapse rate or disability progression at any dose	NCT02975349
Evobrutinib	II	Merck KGaA, Darmstadt, Germany	Completed		RRMS	Annualized relapse rate (ARR)	No—failed to reduce ARR in people with MS	N/A	N/A	NCT04338061
Fenebrutinib	II	Roche–Genentech, Basel, Switzerland	Ongoing Trial		RMS, PPMS	New gadolinium (Gd)-enhancing T1 lesion rate observed on magnetic resonance imaging (MRI) scans of the brain over 12 weeks	Yes	New or enlarging T2-weighted lesion rate observed on MRI scans of the brain over 12 weeks	Yes	NCT05119569
								Proportion of participants free from any new Gd-enhancing T1 lesions and new or enlarging T2-weighted lesions observed on MRI scans of the brain over 12 weeks	Yes	
								Number of participants with adverse events (AEs) and serious adverse events (SAEs)	Not yet—ongoing	
								Number of participants with AEs and SAEs	Not yet—ongoing	
								Number of participants with post-baseline suicidal ideation or suicidal behavior as measured using Columbia Suicide Severity Rating Scale (C-SSRS)	Not yet—ongoing	
Fenebrutinib	III	Roche–Genentech, Basel, Switzerland	Ongoing trial	Study 1: 746 Study 2: 751	RMS compared to teriflunomide	Annualized relapse rate (ARR)	Not yet—ongoing	Time-to-onset of composite 12-week confirmed disability progression (cCDP12)	Not yet—ongoing	NCT04586010 NCT04586023
								Time-to-onset of composite 24-week confirmed disability progression (cCDP24)	Not yet—ongoing	
								Time-to-onset of 12-week confirmed disability progression (CDP12)	Not yet—ongoing	
								Time-to-onset of 24-week confirmed disability progression (CDP24)	Not yet—ongoing	
								Total number of T1 gadolinium-enhancing (Gd+) lesions, new and/or enlarging T2-weighted lesions as detected by magnetic resonance imaging (MRI)	Not yet—ongoing	
								Percentage change in total brain volume from week 24 as assessed by MRI	Not yet—ongoing	
								Change in participant-reported physical impacts of multiple sclerosis (MS) measured by the Multiple Sclerosis, 29-Item [MSIS-29] Physical Scale	Not yet—ongoing	
								Time-to-onset of 12-week confirmed 4-point worsening in Symbol Digit Modality Test (SDMT) Score	Not yet—ongoing	
								Change from baseline to week 48 in the concentration of serum neurofilament light chain (NfL)	Not yet—ongoing	
								Percentage of participants with adverse events (AEs)	Not yet—ongoing	
								Plasma concentrations of fenebrutinib at specified timepoints	Not yet—ongoing	
Fenebrutinib	III	Roche–Genentech, Basel, Switzerland	Ongoing trial	985	PPMS compared to Ocrevus	Time-to-onset of composite 12-week confirmed disability progression (cCDP12)	Not yet—ongoing	Time-to-onset of composite 24-week CDP (cCDP24)	Not yet—ongoing	NCT04544449
								Time-to-onset of 12-week CDP (CDP12)	Not yet—ongoing	
								Time-to-onset of 24-week CDP (CDP24)	Not yet—ongoing	
								Percentage change in total brain volume assessed by magnetic resonance imaging (MRI)	Not yet—ongoing	
								Change from baseline in participant-reported physical impacts of multiple sclerosis (MS) measured by the Multiple Sclerosis Impact Scale, 29-Item [MSIS-29] Physical Scale	Not yet—ongoing	
								Time-to-onset of 12-week confirmed 4-point worsening in Symbol Digit Modality Test (SDMT) Score	Not yet—ongoing	
								Percentage of participants with adverse events (AEs)	Not yet—ongoing	
								Plasma concentrations of fenebrutinib at specified timepoints	Not yet—ongoing	
								Percent change from screening in serum neurofilament light chain (NfL) levels	Not yet—ongoing	
Orelabrutinib	II	InnoCare/Biogen, Beijing, China	Ongoing trial	160	RRMS	The cumulative number of new GdE T1 MRI brain lesions	Yes	Incidence of treatment-emergent adverse events	Not yet—ongoing	NCT04711148
Remibrutinib	III	Novartis, Basel, Switzerland	Ongoing trial	REMODEL:800 (estimated)	RMS	Annualized relapse rate (ARR) of confirmed relapses	Not Yet—ongoing	Time to 3-month confirmed disability progression (3mCDP) on Expanded Disability Status Scale (EDSS)	Not yet—ongoing	NCT05156281
								Time to 6-month confirmed disability progression (6mCDP) on EDSS	Not yet—ongoing	
								Annualized rate of new or enlarging T2 lesion	Not yet—ongoing	
